# Nanomaterials with Glucose Oxidase-Mimicking Activity for Biomedical Applications

**DOI:** 10.3390/molecules28124615

**Published:** 2023-06-07

**Authors:** Shengyi Min, Qiao Yu, Jiaquan Ye, Pengfei Hao, Jiayu Ning, Zhiqiang Hu, Yu Chong

**Affiliations:** State Key Laboratory of Radiation Medicine and Protection, School of Radiation Medicine and Protection, School for Radiological and Interdisciplinary Sciences (RAD-X), Collaborative Innovation Center of Radiation Medicine of Jiangsu Higher Education Institutions, Soochow University, Suzhou 215123, China; 2009401036@stu.suda.edu.cn (S.M.); 2014401021@stu.suda.edu.cn (Q.Y.); 2115408158@stu.suda.edu.cn (J.Y.); 20224220027@stu.suda.edu.cn (P.H.); 20214220030@stu.suda.edu.cn (J.N.); 20215247019@stu.suda.edu.cn (Z.H.)

**Keywords:** nanomaterials, glucose oxidase, nanozymes, activity regulation, biomedical applications

## Abstract

Glucose oxidase (GOD) is an oxidoreductase that catalyzes the aerobic oxidation of glucose into hydrogen peroxide (H_2_O_2_) and gluconic acid, which has been widely used in industrial raw materials production, biosensors and cancer treatment. However, natural GOD bears intrinsic disadvantages, such as poor stability and a complex purification process, which undoubtedly restricts its biomedical applications. Fortunately, several artificial nanomaterials have been recently discovered with a GOD-like activity and their catalytic efficiency toward glucose oxidation can be finely optimized for diverse biomedical applications in biosensing and disease treatments. In view of the notable progress of GOD-mimicking nanozymes, this review systematically summarizes the representative GOD-mimicking nanomaterials for the first time and depicts their proposed catalytic mechanisms. We then introduce the efficient modulation strategy to improve the catalytic activity of existing GOD-mimicking nanomaterials. Finally, the potential biomedical applications in glucose detection, DNA bioanalysis and cancer treatment are highlighted. We believe that the development of nanomaterials with a GOD-like activity will expand the application range of GOD-based systems and lead to new opportunities of GOD-mimicking nanomaterials for various biomedical applications.

## 1. Introduction

**Figure 1:**
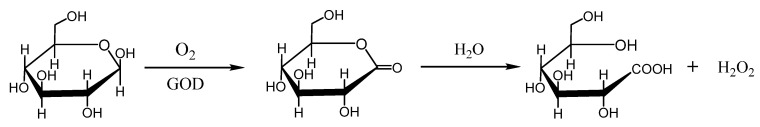
Aerobic glucose oxidation under the catalysis of GOD.

As an oxidoreductase extracted from certain species of insects and fungi (e.g., *Aspergillus niger*), glucose oxidase (GOD) catalyzes the oxidation of glucose into hydrogen peroxide (H_2_O_2_) and D-glucono-δ-lactone (Figure 1) [1]. To date, GOD has been widely used in the field of vegetal raw materials production, the food industry and biomedicine. For instance, the fast determination of blood glucose levels is mainly performed by GOD-based biosensors [2]. In addition, the important industrial raw material gluconic acid or gluconate is mainly produced by the catalytic oxidation of GOD into glucose [3]. Although natural GOD possesses a high catalytic activity, it has intrinsic disadvantages, including being difficult to purify and easy to inactivate [4]. To overcome the aforementioned drawbacks, it is highly desirable to discover GOD alternatives for biomimetic catalytic oxidation of glucose.

In the 1990s, the glucose oxidation catalyzed by transition metal nanomaterials was developed for the industrialized production of gluconic acid [5]. However, such catalysts were often toxic and biologically incompatible. Moreover, their optimal catalytic temperature was much higher than the human body temperature, making them impossible to be used in biomedical applications. Recently, numerous nanomaterials have been reported with enzyme-mimetic characteristics, which present unique catalytic functions toward specific biomolecules under physiological conditions [6,7,8,9]. With the rapid progress of nanotechnology and nanozymes, various nanomaterials with an GOD-like activity were continuously discovered to mimic the function of GOD [10]. Compared to natural GOD, GOD-mimicking nanomaterials have an adjustable catalytic efficiency, a high stability and a large-scale production, exhibiting broad application prospects. Despite the remarkable advances that have been made, no comprehensive review has been devoted to GOD-mimicking nanozymes, and a deep understanding of GOD-mimicking nanomaterials, as well as their biomedical applications, is urgently needed.

Therefore, in this review, we summarize the discovery, catalytic mechanism, activity regulation and representative biomedical applications of GOD-mimicking nanozymes and prospect their future development. First, the representative GOD-mimicking nanomaterials and their proposed catalytic mechanisms are depicted. Then, the efficient modulation strategy for improving the catalytic activity of existing GOD-mimicking nanomaterials is introduced. Finally, the potential biomedical applications in glucose detection, DNA bioanalysis and cancer treatment are highlighted.

## 2. Nanomaterials with GOD-like Activities

The development of nanomaterials to simulate GOD has become a research hotspot owing to the inherent drawbacks of natural GOD. To date, various Au-based nanoparticles (NPs), as well as other nanomaterials, MnO_2_ NPs for instance, have been reported to mimic the function of GOD [11,12]. In this section, we will illustrate in detail the current nanomaterials with a GOD-like activity. Their reaction kinetics and catalytic mechanisms will also be introduced below.

### 2.1. Au Nanomaterials

Early in 2004, the Massimiliano Comotti team first discovered that “naked” Au NPs catalyzed the aerobic oxidation of glucose at room temperature [13]. Shortly after, several Au-based nanomaterials were developed as analogs of natural GOD, which stimulated the research of GOD-mimicking nanozymes and their biomedical applications [14,15,16].

**Figure 2:**
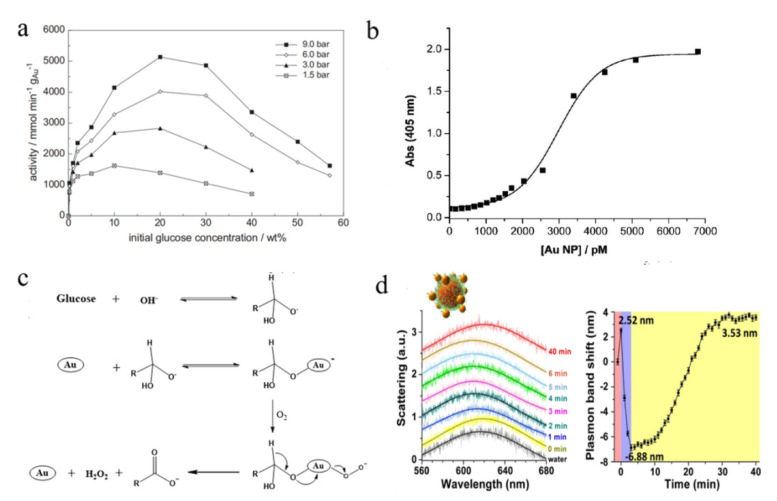
Kinetics and reaction mechanisms of glucose oxidation catalyzed by Au nanomaterials. (**a**) Effect of oxygen partial pressure and initial glucose concentration on the catalytic activity of Au NPs toward glucose. Reprinted with permission from Ref. [17]. Copyright 2011, Elsevier. (**b**) Effect of Au nanozyme on the turnover of glucose. Reprinted with permission from Ref. [11]. Copyright 2010, American Chemical Society. (**c**) Catalytic mechanism of Au NPs on the aerobic oxidation of glucose. Reprinted with permission from Ref. [18]. Copyright 2006, John Wiley & Sons. (**d**) Quantitative determination of a single Au NP catalysis toward glucose oxidation. Reprinted with permission from Ref. [20]. Copyright 2015, American Chemical Society.

As a metal nanocatalyst, the reaction kinetics of Au NPs toward glucose oxidation conform to the Langmuir–Hinshelwood model and the apparent activation energy is approx. 54 kJ mol^−1^ [17]. The oxidation rate of glucose catalyzed by Au NPs has a pronounced dependency on the substrate concentration (initial glucose concentration and dissolved oxygen concentration). For instance, the catalytic activity of Au NPs shows a positive correlation trend with the initial glucose concentration below 20% wt, which is gradually decreased with the increase in the glucose concentration when it is higher than 30% wt (Figure 2a) [17]. Similarly, there is a positive effect of the dissolved oxygen concentration on the glucose oxidation rate and the highest catalytic activity is achieved at 4 mM of dissolved oxygen. In addition, the reaction turnover of glucose into gluconic acid is regulated by various parameters of Au NPs, including the concentration, size, shape and surface passivation. For citrate-capped Au NPs, the catalytic activity increases with the Au NPs content below 2 nM and it reaches a plateau at 4 nM (Figure 2b) [18]. However, when Au NPs are increased from 13 nm to 30–40 nm by the Au NPs-based self-catalyzed system, their GOD-mimicking activity is almost completely blocked owing to the enlarged size and the surface passivation of Au NPs.

Similar to natural GOD, Au NPs catalyze the glucose oxidation by the dehydrogenation of glucose and the reduction in O_2_ through a two-electron mechanism to produce gluconate and hydrogen peroxide (H_2_O_2_) (Figure 2c) [13]. First, the glucose molecules are transformed into hydrated glucose anions using OH^−^ in an aqueous solution since the Brønsted base can abstract H^+^ from glucose. Then, the hydrated glucose molecules are easily adsorbed on the surface of the Au NPs and the generated negatively charged Au–glucose complex can activate the dissolved O_2_ through the nucleophilic addition reaction to produce Au–peroxy intermediates. Eventually, these intermediates undergo negative hydrogen migration and rearrangement to obtain H_2_O_2_ and gluconate. It’s worth noting that part of the reaction product H_2_O_2_ will decompose before reaching the critical concentration of the glucose oxidation and will act as a possible competitor for O_2_ in glucose oxidation [18]. Interestingly, other noble metal NPs (i.e., Pt, Pd, Ru, Rh and Ir) were also discovered to catalyze the oxidation of glucose, except that O_2_ is directly reduced to H_2_O using the four-electron process [19].

In order to in situ monitor the surface catalysis of a single Au nanoparticle toward glucose oxidation, Fan and his colleagues designed Au nanohalo structures by coupling GOD-mimicking small Au NPs with plasmonic large Au NPs [20]. The proximity between the two types of Au NPs resulted in a locally enhanced electromagnetic field, which was extremely sensitive to the catalytic reaction occurring on the surface of the Au nanohalo. As shown in Figure 2d, the addition of glucose led to an initial 2.52 nm redshift due to the adsorption of glucose on the Au nanohalo (approx. 302 glucose molecules per nanohalo). The following blue shift of 6.88 nm and redshift of 3.53 nm, corresponding to the charging and discharging processes, were attributed to the efficient transfer electrons (approx. 66 electrons per second) between the adsorbed glucose and O_2_. The retarded discharging mainly resulted from the reduction in dissolved oxygen. These pioneering works displayed the catalytic process of Au NPs in glucose oxidation and laid the foundation for the development of various GOD-mimicking Au-based nanomaterials.

It has been known that Au NPs are highly effective in simulating natural GOD to catalyze the aerobic oxidation of glucose. However, the unsupported Au NPs are easy to agglomerate and inactivate in complex bioenvironments, which will significantly reduce their GOD-like activity. In addition, sole Au NPs are unable to simulate coupled multi-enzyme systems to achieve biomimetic cascade catalysis. Therefore, significant varieties of nanocarriers have been developed to support or encapsulate the GOD-mimicking Au NPs to hinder their aggregation and maintain the unflinching catalytic performance toward glucose [21,22]. The current reported nanocarriers of GOD-mimicking Au NPs are summarized in Table 1 for reference.

**Figure 3:**
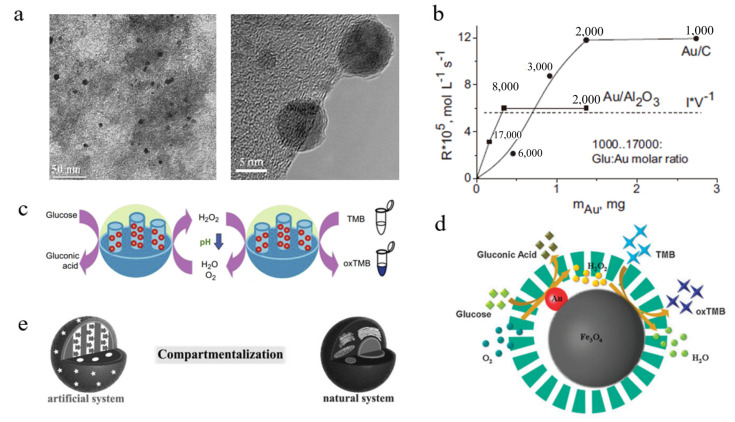
Au-based nanocomposites for GOD-like biomimetic catalysis. (**a**) TEM images of Au NPs supported on activated carbon. Reprinted with permission from Ref. [24]. Copyright 2012, Elsevier. (**b**) Reaction rate of glucose oxidation catalyzed by Au/Al_2_O_3_ or Au/C nanocomposites. Reprinted with permission from Ref. [26]. Copyright 2013, Elsevier. (**c**) Bioinspired tandem catalysis by dual enzyme-like Au NPs supported within mesoporous silica. Reprinted with permission from Ref. [35]. (**d**) Bioinspired cascade system of Fe_3_O_4_-supported Au microspheres encapsulated in mesoporous silica shells. Reprinted with permission from Ref. [25]. Copyright 2013, RSC Publishing. (**e**) Compartmentalization of GOD-mimicking Au NPs and POD-mimicking hemins in in mesoporous silica-based nanoreactors. Reprinted with permission from Ref. [33]. Copyright 2016, John Wiley & Sons.

Among the various nanocarriers, carbon-based materials have been widely used as industrial catalysts or metal catalyst supports [31,32]. Inspired by this feature, the activated carbon-supported Au nanozyme was designed using plasma reduction for the selective oxidation of glucose (Figure 3a) [24]. As a result, the carbon support improves the dispersion of the Au NPs and is conducive to the effective collision between the glucose molecules and the GOD-mimicking Au NPs in the aqueous phase, thereby effectively improving the catalytic activity toward glucose.

In addition to activated carbon, other carbon nanomaterials (e.g., graphene) and engineered oxide nanomaterials (e.g., Al_2_O_3_, SiO_2_) were also developed as nanocarriers for Au NPs-based biomimetic catalysis [25,26,27,33]. It should be noted that these nanocarriers not only help the formation of well-dispersed Au NPs but also determine the overall reaction kinetics by affecting the diffusion and transport of dissolved oxygen and substrate glucose. For instance, the oxidation rate of glucose over Au/Al_2_O_3_ nanocomposites is higher than over Au/C catalysts at high glucose:Au ratios, while the catalytic efficiency of Au/Al_2_O_3_ is much lower than Au/C at low glucose:Au ratios (Figure 3b) [26]. This phenomenon can be partly ascribed to the facilitating oxygen dissolution and transfer by the hydrophobic carbon support. Mesoporous silica nanomaterial (MSN) is another model support for embedding Au NPs-based nanozymes to realize a higher GOD activity [27]. On the one hand, the MSN maintains an extraordinary stability of small and well-dispersed Au NPs under different physiological and pathological conditions. On the other hand, the mesoporous structure allows the small glucose molecules and O_2_ to freely diffuse between the outer surface and the inside pores.

Apart from the optimized dispersity and reaction kinetics, the nanocarrier-supported Au NPs have also been discovered with dual enzyme-like catalytic activities for realizing the biomimetic enzymatic cascade reaction [34]. By taking advantage of the GOD-like activity, the obtained MSN–Au NPs catalytically oxidize glucose to yield gluconic acid and H_2_O_2_ in the physiological environment (Figure 3c) [35]. The generated gluconic acid gradually decreases the ambient pH, which activates the peroxidase (POD)-like activity of the Au NPs. As a result, the other product H_2_O_2_ is simultaneously reduced by the MSN–Au NPs in the presence of tetramethylbenzidine (TMB). Therefore, the MSN–Au NPs present a GOD-activated dual nanozyme-catalyzed cascade reaction.

Given the weak POD-like activity of Au NPs, the GOD-mimicking Au NPs can be further assembled using natural POD or other POD-mimicking nanomaterials to improve the catalytic efficiency of the artificial cascade catalytic systems. For instance, Au NPs and natural hemin were co-assembled in supramolecular nanostructures to mimic the glucose conversion cascade reaction [33]. In addition, ultra-fine Au NPs with a high GOD-like activity were directly supported on the POD-mimicking Fe_3_O_4_ cores to construct Fe_3_O_4_–Au microspheres with both high GOD- and POD-like activities (Figure 3d) [25]. Furthermore, in order to avoid mutual interference between the different catalytic reactions, each enzyme or nanozyme can be positioned at a spatially separate domain within the nanocarriers to mimic the compartmentalization process of the tightly controlled cellular compartments. For instance, a multilayer polyelectrolyte-coated MSN has been proven to achieve a compartmentalization of GOD- and POD-like nanozymes to mimic the complicated enzymatic reactions in the cell organelles (Figure 3e) [33]. Among this nanoreactor, the GOD-mimicking Au NPs are assembled inside the channel of the MSN mesoporous silica while hemins with a POD-like activity are decorated on the surface polyelectrolyte. As a permeable membrane, the polyelectrolyte multilayers enable the small molecules to flux freely from the nanoreactor. The tandem catalytic process plays an important role for GOD-mimicking Au nanomaterials in biosensing and biomedicine applications.

In addition to Au-based noble metal nanomaterials, other nanostructures, such as metal oxides and nitrogen carbide (C_3_N_4_) nanomaterials, have also been discovered to possess a GOD-like activity with the advantages of low prices, high activities and scaled production, which are expected to be promising alternatives for natural GOD [12,36].

### 2.2. MnO_2_ Nanomaterials

**Figure 4:**
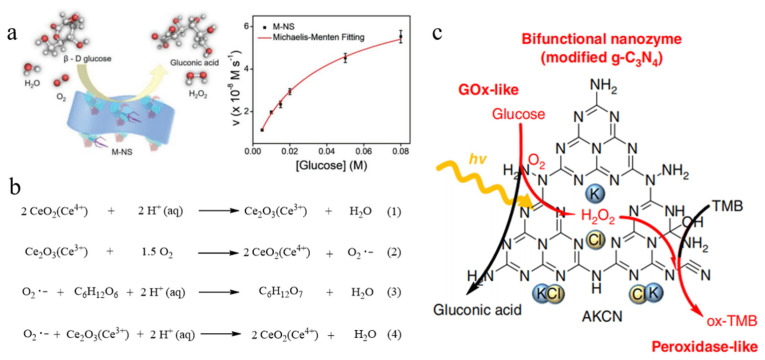
Au-free nanomaterials as GOD-like nanozymes. (**a**) Glucose depletion catalyzed by BSA-directed MnO_2_ nanoflakes and the corresponding kinetics curve. Reprinted with permission from Ref. [12]. Copyright 2019, John Wiley & Sons. (**b**) Catalytic mechanism of nanoceria on aerobic oxidation of glucose. Reprinted with permission from Ref. [38]. (**c**) Schematic illustration of photocatalytic aerobic oxidation of glucose using modified g-C_3_N_4_ for glucose detection. Reprinted with permission from Ref. [36].

Although MnO_2_ nanomaterials have been widely reported to mimic POD- and catalase (CAT)-like activities, their GOD-mimicking property has rarely been reported during the past years. In 2018, BSA-directed MnO_2_ nanoflakes were first discovered with an inherent catalytic property toward the aerobic oxidation of glucose (Figure 4a) [12]. The remarkable concentration-dependent GOD-like activity mainly originates from MnO_2_ nanostructures instead of leached Mn ions because the high concentration of free Mn ions presents a negligible GOD-like activity. The steady-state enzyme kinetics analysis demonstrates that MnO_2_ nanoflakes have much higher affinity for glucose than natural GOD [37]. It is worth noting that the dosage of templated BSA can optimize the GOD-like activity of MnO_2_ nanoflakes by modulating their size and thickness.

Recently, other MnO_2_ nanomaterials with mixed components or different morphologies were also reported with a strong GOD-like activity. For instance, nanolayered MnCaO_2_ is able to catalytically oxidize glucose and produce gluconic acid [39]. Similarly, ultra-small BSA-coated MnO_2_ nanodots can convert glucose and O_2_ into gluconic acid and H_2_O_2_ [12]. The fascinating GOD-mimicking property stems from their abundant exposed active sites and is enhanced by their satellite-like BSA corona. Given the widely reported POD- and CAT-like activities of the Mn-based NPs, the GOD-mimicking MnO_2_ nanomaterials have demonstrated a prominent superiority in cascade catalysis-based biological applications.

### 2.3. CeO_2_ Nanomaterials

As another representative metal oxide nanomaterials, nanoceria have been explored to catalytically oxidize a diverse range of substrates, such as TMB, POD and catechol [38]. In 2020, the GOD-like activity of well-dispersed CeO_2_ nanomaterials were reported and the possible catalytic mechanism of glucose oxidation was examined in detail (Figure 4b) [27]. In brief, Ce(IV) on the surface of CeO_2_ NPs can be partly reduced into Ce(III) by using hydrogen ions in an aqueous solution. The generated Ce(III) or oxygen vacancy in nanoceria can react with the adsorbed O_2_ to produce Ce(IV) and reactive superoxide anion radicals, which catalyze the oxidation of glucose to gluconic acid. Therefore, the automatic redox switching of the two oxidation states (Ce(III) and Ce(IV)) on the surface of nanoceria plays a very important role in the nanoceria-initiated catalytic oxidation toward glucose [38].

Inspired by the GOD-like activity of small-sized nanoceria, several CeO_2_-based nanocomposites with different nanostructures, such as porous CuO–CeO_2_ nanospheres, CeO_2_@MnO_2_ core–shell heterojunctions and CeO_2_-encapsulated Ag–Au nanocages, were successively developed as novel GOD-mimicking nanozymes [40,41,42]. For instance, porous CuO–CeO_2_ nanospheres not only provide additional oxygen vacancies at the Cu–Ce interfaces but also afford a high oxygen mobility owing to the porous structure, which ensures the nanospheres exhibit an extra strong GOD-like activity [40]. Zhang and his colleagues integrated ultra-small CeO_2_ NPs within an Ag–Au nanocage to promote the stability and catalytic activity of GOD-mimicking nanoceria [42]. These CeO_2_-based nanomaterials with a GOD-like activity will help to construct non-enzymatic systems for biomedicine and biomimetic catalysis.

### 2.4. C_3_N_4_ Nanomaterials

With the discovery of various GOD-mimicking nanozymes, the metal-free artificial enzyme that functions similar to GOD is highly desirable. In 2019, it was first reported that graphitic carbon nitride (g-C_3_N_4_) could catalyze glucose oxidation and concurrent two-electron O_2_ reduction under visible light (Figure 4c) [37]. However, pure g-C_3_N_4_ suffers from a low photocatalytic efficiency toward glucose oxidation. To improve the photo-excited GOD-like activity, Choi et al. designed modified g-C_3_N_4_ using a calcination procedure with KOH and KCl, which presented a significantly enhanced H_2_O_2_ production and an accelerated gluconic acid conversion from glucose oxidation under visible light irradiation [37]. The improvement of the charge separation and formation of the charge delivery channels in modified g-C_3_N_4_ facilitated the directional charge transfer and were critical for the GOD-mimicking behavior. The photocatalytic property of modified g-C_3_N_4_ is specific to glucose and is not active to other glucose analogues. In addition, the modified g-C_3_N_4_ exhibits an intrinsic POD-like activity under the dark condition, motivating its application for real-time monitoring of glucose in a sequential light–dark process.

On the basis of the GOD-mimicking behavior of C_3_N_4_-based nanomaterials, Sarkar et al. synthesized KCl-fused g-C_3_N_4_ with 3D mesoporous flower-like morphology, which ensured their superior GOD-like activity since the abundant modified g-C_3_N_4_ nanosheets were randomly arranged in a porous structure [43]. The 3D g-C_3_N_4_ could be further assembled using POD-mimicking chitin–AcOH to achieve a super-sensitive colorimetric detection of H_2_O_2_ and glucose in real human serum and urine samples. The discovery of the metal-free nanozyme system enriches the GOD-mimicking nanomaterials and can be successfully applied in medical diagnoses and treatment.

### 2.5. Others

In addition to the above-mentioned nanomaterials, several other types of nanostructures have also been reported to possess a GOD-like activity. For instance, intermetallic phase Pd_x_Te_y_ catalysts present both a high activity and a high selectivity to the catalytic oxidation of glucose and their GOD-like activity can be optimized by altering the content of Pd and Te [44]. V_2_O_5_ nanobelts were discovered to possess an intrinsic catalytic activity for glucose oxidation because the glucose molecules can be preferentially adsorbed on the V_2_O_5_ plane [45]. The alkali-soaking cobalt metal–organic framework (MOF) is able to catalyze the reaction between glucose and O_2,_ owing to the presence of a Co_x_O_y_H_z_ active center [46]. Recently, transition metal phosphides, FeP, CoP, Ni_2_P and Cu_3_P were reported to have diverse enzyme-like activities and only FeP catalyzed the dehydrogenation of glucose into gluconic acid [47]. In a word, various GOD-mimicking nanomaterials have been gradually discovered while the rational design and in-depth understanding of the catalytic process toward glucose is still in its infancy.

## 3. Activity Regulation of GOD-mimicking Nanomaterials

In contrast to natural GOD, the GOD-like activity of nanozymes can be finely modulated by regulating the physicochemical properties of the nanomaterials. Therefore, to optimize the catalytic performance of various nanomaterials toward glucose, the typical physicochemical parameters of GOD-mimicking nanomaterials, including the size, morphology, composition and surface modification have been well engineered. In addition, the surrounding environments, such as the pH, temperature and light illumination, also greatly affect the catalytic performance of the GOD-mimicking nanomaterials.

### 3.1. Impact of the Physicochemical Parameters in the Catalytic Performance of Nanomaterials

#### 3.1.1. Size and Morphology

**Figure 5:**
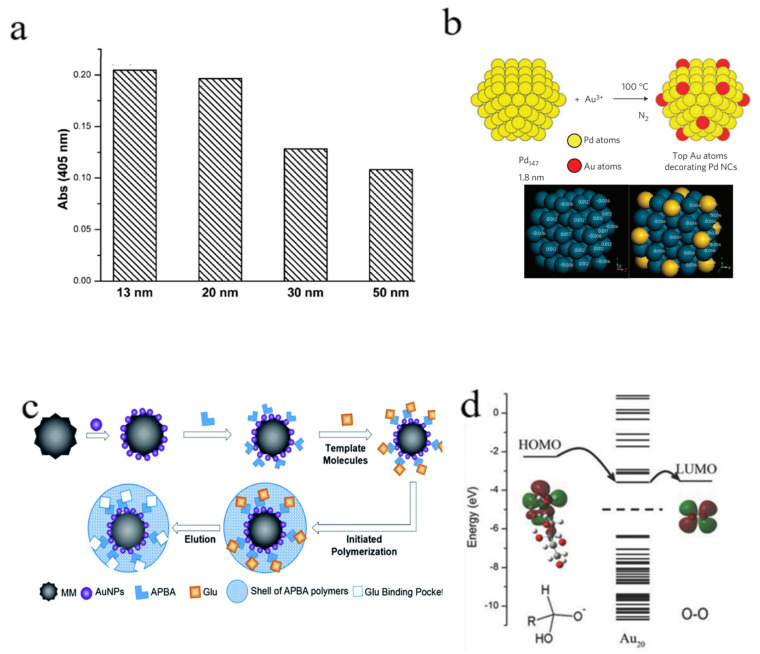
Activity regulation of GOD-mimicking nanomaterials. (**a**) Size-dependent catalytic activity of Au NPs toward aerobic glucose oxidation. Reprinted with permission from Ref. [11]. Copyright 2010, American Chemical Society. (**b**) Effect of the Au atoms position on the GOD-like activity of metal alloy nanomaterials. Reprinted with permission from Ref. [54]. Copyright 2011, Springer. (**c**) GOD biomimetic Au–Pt NPs with a high glucose selectivity using molecular imprinting. Reprinted with permission from Ref. [58]. Copyright 2019, RSC Publications. (**d**) Scheme of the hot electron-induced charge transfer for the enhanced GOD-like activity of Au NPs. Reprinted with permission from Ref. [61]. Copyright 2018, John Wiley & Sons.

The size and morphology of nanomaterials is of great significance for their catalytic and GOD-like activity. Generally, the GOD-like activity of nanomaterials is size-dependent and smaller-sized nanomaterials present a higher catalytic activity on glucose oxidation due to the presence of a larger specific surface area. For instance, the catalytic efficiency of Au NPs toward glucose oxidation is inversely related to their diameters within the limits of 13–50 nm, which is attributed to the change in the specific surface area of Au NPs (Figure 5a) [11]. Similarly, Zhang et al. reported that the GOD-like activity of Au NPs declined as their size increased from 5 to 60 nm. By taking advantage of this phenomenon, Fan et al. fabricated plasmonic large Au NPs with GOD-mimicking small Au NPs to achieve the sensitive detection of the glucose catalytic process [11]. Consistently, the GOD-like activity of nanoceria increased with a decrease in the particle size. Recently, Chen and his colleagues carried out the protein-directed synthesis of 2D MnO_2_ nanosheets with a controllable size and thickness [12]. With an increase in the BSA content from 0.1- to 1- and 10-fold, the diameter of the MnO_2_ nanosheets decreased from 295.9 nm to 105.4 nm, and then increased to 343.9 nm. Interestingly, the small-sized 2D MnO_2_ nanosheets directed by a moderate BSA dosage (one-fold) presented the optimal GOD-like activity, which was consistent with the previous report of the GOD-mimicking nanomaterials. Notably, with the size reduction of nanozymes to the level of a single atom, the catalytic reactions were carried out on a single atom to achieve a higher catalytic efficiency [48]. For instance, Rh single-atom nanozymes were found to display a stronger GOD-like activity than natural GOD in both the biometabolism and electrometabolism of glucose oxidation under neutral conditions, owing to the maximized atomic utilization efficiency [49].

In contrast with the clear size effect, the morphology of GOD-mimicking nanomaterials on the glucose catalytic oxidation has rarely been reported. In 2021, Kim et al. synthesized novel Au NPs with a unique rhombic dodecahedron morphology using a gallnut extract as the reducing and stabilizing agent [50]. Compared to the traditional spherical shape or clusters of Au NPs, the gallnut extract-capped Au NPs exhibited a higher GOD-like activity owing to the unique rhombohedral dodecahedron morphology. The enhanced GOD activity was mainly attributed to the increased catalytic sites on the dodecahedron surface of the Au NPs.

#### 3.1.2. Composition

The activity of GOD-mimicking nanozymes is highly related to the composition of nanomaterials. Therefore, tuning the composition of GOD-mimicking nanomaterials, such as the construction of composite nanomaterials with an alloy structure or heteroatom doping, is another attractive strategy for optimizing the catalytic oxidation process toward glucose.

It has been widely accepted that bimetallic alloys or core–shell nanozymes have a superior catalytic activity compared to individual metal counterparts, owing to the adjustment of the surface strain and electronic coupling between the constituent atoms. For example, Au NPs can be alloyed with other metals, such as Pt, Pd and Ag atoms, to improve the reaction activity [51,52,53]. Compared to the Au NPs counterparts, bimetallic NPs with different composite ratios presented a significantly improved GOD-like activity. Among these Au-based nanocomposites, the Au–Pt alloy nanomaterials show the best catalytic performance toward glucose. In addition, the ratio of Au to Pt also affected the catalytic activity in the Au–Pt alloy [52]. With the increase in Au:Pt, the catalytic activity increased first and then decreased, reaching the best activity at Au:Pt = 1. Similarly, the introduction of Te on Pd NPs enhanced the conversion degree and selectivity of Pd NPs in the catalytic oxidation of glucose and the nanocomposites with a small amount of Te (below 5%) exhibited the best GOD-like activity [44].

The difference in glucose catalytic oxidation using GOD-mimicking nanozymes not only stems from the elemental composition but also is highly related to the structure of the nanocomposites. For instance, Au–Ag core–shell nanostructures have a stronger GOD-like activity than the Au–Pt core–shell counterparts, but present a significantly lower catalytic activity than the Au–Pt alloy composites with the same atom ratio and diameter [54]. To elucidate the critical role of the metal atoms location on the GOD-like activity, Toshima and colleagues synthesized a crown-jewel-structured Au/Pd nanocluster with different Au contents [55]. As shown in Figure 5b, the Au atoms were controllably decorated at the top sites of the Pb clusters, which imparted the Au/Pd nanocluster with the maximum GOD-like activity (approx. 194,980 moles glucose h^−1^ per mole Au), which was much higher than the monometallic formulations (Au or Pb nanoclusters) and Au/Pd alloy nanoclusters. The deep understanding of the composition activity relations at the atomic level may accelerate the commercial exploitation of GOD-mimicking nanomaterials.

For non-metallic nanomaterials, such as the aforementioned C_3_N_4_, heteroatom modification is a more common method, which can improve its catalytic efficiency of glucose oxidation. To improve the photo-driven glucose oxidation on C_3_N_4_, Choi et al. modified C_3_N_4_ with a variety of elements containing alkali metal ions and halide ions [37]. In comparison to the other counterparts, C_3_N_4_ incorporated with KOH and KCl exhibited the highest GOD-mimetic activity and H_2_O_2_ production as the modification of KCl/KOH facilitated the charge separation and transfer while the charge recombination was hindered, thereby prolonging the lifetime of the photogenerated charge carriers.

#### 3.1.3. Surface Modification

Surface ligand modification can also significantly affect the catalytic activity of GOD-mimicking nanomaterials since it can regulate the binding relationship of the substrates and nanozymes. According to the elementary reaction theory, the reaction involving enzyme requires an effective collision between the substrates and enzymes. The ligand modification on the nanozymes will influence this collision process between the catalytic sites and substrates, especially the glucose molecules, and then change the catalytic reaction rate. For instance, the adsorption of small molecules such as citrate on the Au NPs does not deactivate the Au NPs, while the adsorption of polymers, including serum proteins and high molecular weight polymers, inhibits glucose catalytic oxidation [56]. For the MnO_2_ NPs, the amount of BSA significantly affects the GOD-like activity of the MnO_2_ NPs, showing a trend of promotion at a low concentration and inhibition at a high concentration [57].

Interestingly, specific surface modification on the GOD-mimicking nanozymes, such as molecular printing technology or chiral recognition catalysis, can realize the glucose recognition for selective catalysis. By using molecular imprinted polymers to simulate the interaction between nanozyme and substrate glucose, Zhang and his group prepared Au–Pt alloy NPs and the binding sites for glucose were designed using molecular imprinting, which greatly improved the catalytic activity and selectivity of glucose (Figure 5c) [58]. The molecularly imprinted polymer shells with specific glucose binding pockets were constructed using glucose bindable aminophenylboronic acid to improve the affinity to glucose, resulting in a catalytic efficiency approx. 200 times higher than that of the Au NPs. More importantly, the Au–Pt alloy nanozymes had no binding pockets to capture other saccharides except for glucose [58]. In addition to saccharides species selectivity, GOD-mimicking nanozymes modified with chiral ligands can selectively recognize chiral glucose for enantioselective catalysis. For instance, Au NPs capped with chiral phenylalanine present a selective adsorption towards D- and L-glucose [59]. Therefore, L-phenylalanine-capped Au NPs preferentially catalyze the D-glucose oxidation, while D-phenylalanine-capped Au NPs demonstrate a higher catalytic activity toward L-glucose. The DNA-guided biomimetic chiral catalysis of glucose oxidation also has been reported. For instance, the Au NPs decorated with random-coiled DNA prefer to adsorb L-glucose and the structured DNA-capped Au NPs prefer to adsorb D-glucose, further realizing the chiral selectivity of Au NPs toward the catalytic oxidation of glucose enantiomers [60].

### 3.2. Impact of the Environmental Parameters in the Catalytic Performance of Nanomaterials

#### 3.2.1. Environmental pH and Temperature

In the process of glucose oxidation catalyzed by GOD-mimicking nanozymes, the temperature and pH have a significant influence on the catalytic process. According to the Arrhenius equation, there is a significant correlation between the activation energy of the enzyme and the ambient temperature. However, the optimal temperature should not be too high for the natural GOD because the stability of the natural enzyme is extremely sensitive to temperature changes. In contrast, GOD-mimicking nanozymes present a much better stability than natural GOD. For instance, MnO_2_ nanomaterials retained more than 70% of the initial GOD-like activity after incubation at a temperature ranging from 4 °C to 90 °C, while the activity of natural GOD remarkably decreased to as low as 19% [11,57].

The pH is another important environmental parameter for the catalytic oxidation reaction of glucose as the hydrogen transfer is involved in the reaction process. The change in the pH can significantly change the redox potential of the reactants and then accelerate or inhibit the catalytic reaction. Similar to the effect of the temperature, GOD-mimicking nanozymes such as Au NPs have a stronger stability than natural GOD, whose high catalytic activity can still be maintained in a wide pH range [11,39,57]. Although an excessive acid or base will slightly weaken the catalytic activity, most of the current reported GOD biomimetic nanomaterials show an optimal pH value at approx. 4.0. Therefore, GOD-mimicking nanozymes have a higher thermal and pH stability and catalytic endurance for aerobic glucose oxidation.

#### 3.2.2. Light Illumination

Light regulation is an emerging regulation strategy for the GOD-mimicking reaction. According to the optical quantum theory, different wavelengths of light excite the electronic transition and regulate the energy band gap. Therefore, integrating the light energy and inherent GOD-like activity of biomimetic nanozymes is conducive to the electron transfer in the process of the glucose oxidation reaction, thus leading to the improvement of the GOD-like activity [41,61]. The previous studies mainly focused on the photothermal conversion induced by hot electrons and the direct charge transfer to the adsorbed reactants. For instance, by incorporating the GOD-like activity and surface plasmon resonance (SPR) of the Au NPs, hot electrons generated from the visible light-excited Au NPs accelerated the reaction between the glucose molecules and O_2_ in close proximity, thus enhancing the GOD-like activity of the Au NPs [62].

Recently, further studies showed that the hot holes generated simultaneously with the hot electrons also play an important role in the photo-enhanced GOD biomimetic activity. The hot holes are conducive to the transfer of catalytic intermediates to the product glucose acid molecules, and then desorb from the surface of the nanozymes to restore the GOD-like activity of the nanozymes. For instance, laser illumination improves and reactivates the GOD-like activity of Au NPs and significantly increases the generation of gluconic acid (Figure 5d) [61]. The mechanism analysis demonstrates that the light excitation creates excited hot electrons on the surface of Au NPs that directly participate in the catalytic reduction reaction while vacant hot holes accept electrons from the HOMO of the intermediates. As the adsorption energy of gluconic acid (0.2–0.3 eV) is much lower than intermediate molecules (≈3.0 eV), light excitation favors to desorb from the surface of Au NPs, leading to the reactivation of the GOD biomimetic Au NPs.

## 4. Biomedical Applications of GOD-mimicking Nanomaterials

Due to the advantages of GOD-mimicking nanomaterials compared to natural GOD, such as an intrinsic high catalytic activity, a strong resistance to environmental differences and a satisfactory catalytic stability, GOD-mimicking nanomaterials have been widely used in diverse biomedical applications. In this section, we highlight and discuss the current advancement of GOD-mimicking nanomaterials in biomedical applications from the detection of key biomolecules (e.g., glucose, H_2_O_2_, DNA) to disease treatment.

### 4.1. Glucose Detection

Glucose is the main energy supplier of living organisms and is also a common substance in life. As is already well known, glucose metabolic disorders are highly prevalent diseases worldwide and contribute to the development of various pathological conditions. As a result, glucose testing is widely used in clinical studies and daily life. Currently, the precise detection of glucose is generally divided into two processes, namely the glucose oxidation stage catalyzed by GOD and the chromogenic reaction stage catalyzed by HRP.

**Figure 6:**
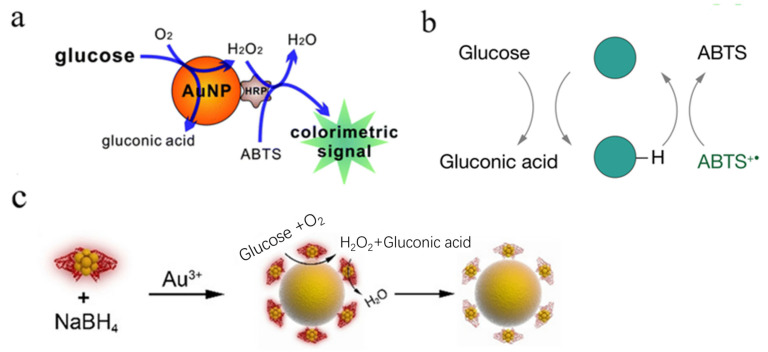
GOD-mimicking nanomaterials for glucose detection. (**a**) Schematic illustration of Au NPs–HRP nanoconjugates for glucose determination. Reprinted with permission from Ref. [63]. Copyright 2012, RSC Publications. (**b**) ABTS^+•^ as an electron acceptor and colorimetric indicator for the one-step detection of glucose. Reprinted with permission from Ref. [19]. (**c**) Schematic diagram of histidine-protected Au NCs-mediated fluorometric detection of glucose based on the self-catalyzed enlargement of Au NPs. Reprinted with permission from Ref. [16]. Copyright 2019, John Wiley & Sons.

Owing to the intrinsic drawbacks of natural GOD, it is promising to design GOD-mimicking nanomaterials for glucose detection. By simulating the classic cascade reaction, Fan and his colleagues coupled Au NPs with HRP as promising nanosensors for efficient glucose determination since the appropriate concentration of HRP does not affect the GOD-like activity of Au NPs (Figure 6a) [63]. Subsequently, Li et al. developed Au NPs with simultaneous GOD-like and HRP-like activities at the same pH, which provided a fast, one-pot glucose colorimetric detection assay [64]. In addition, the introduction of L-cysteine into the system can significantly improve the selectivity of glucose detection, which could be applied to the determination of glucose in human serum [53]. Recently, by utilizing ABTS^+•^ instead of O_2_ as an electron acceptor, Yan and her group realized the rapid one-step colorimetric detection of glucose (Figure 6b) [19]. In the presence of glucose, GOD-mimicking metal NPs catalyzed the reduction of ABTS^+•^ accompanied by a decrease in the absorption at 734 nm. The colorimetric detection of glucose also could be achieved by coupling GOD-mimicking Au NPs with Ag NPs because the catalytically generated peroxide dissolves the Ag NPs, resulting in distinct color changes [16,53].

The traditional colorimetric detection method needs to use the color reaction of TMB, and the steps are relatively cumbersome. In addition to the catalytic activity, the Au NPs also have fluorescent properties, which can be used to achieve the glucose determination with a much lower detection limit. For instance, Au nanoclusters (NCs) with both enzyme-like activities and fluorescent properties could result in TMB oxidation and fluorescence quenching using the catalytic decomposition of the generated H_2_O_2_ [65]. Without the aid of external indicators, a fluorescent AuNP–AuNC nanosystem could be used for glucose determination in one step. Subsequently, histidine modified Au NPs for the fluorescent detection of glucose have also been developed (Figure 6c) [16]. In brief, Au NPs were used as the GOD simulation catalysts for glucose oxidation to generate H_2_O_2_ in situ, and then the produced H_2_O_2_ reacted with HAuCl_4_ to form Au(0) on the surface of the Au NPs, leading to the expansion of the Au NPs and its absorption spectrum change [65]. Since there is a relatively strong overlap between the SPR absorption of the Au NPs (as absorbers) and the fluorescence emission of the histidine-protected Au NCs (as fluorophores), the fluorescence intensity of the Au NCs varies compared to Au NPs. This IFE (inner filter effect)-based analysis showed a good performance in the detection of glucose and the detection limit was 3.4 μM, which was suitable for the highly selective detection of glucose in urine samples of diabetic patients.

Apart from the fluorescence and colorimetric method, there is a new biosensing assay for converting the H_2_O_2_ generated by the oxidation of glucose into electrical signals through the GOD-mimicking activity of nanostructures. For instance, Titanium dioxide (TiO_2_) is an excellent photoelectrode material for photoelectrochemical sensors. The modification of GOD- and HPR- mimicking Co-MOF nanomaterials exhibit a strong photocurrent effect under the excitation of a xenon lamp light source [46]. This glucose detection method improved the selectivity of the glucose determination, and the lowest detection limit reached 0.03 μM.

The rapid and accurate determination of the blood glucose level is one of the most important issues in medical and pharmaceutical research, particularly for diagnosing diabetes mellitus. Developing a fast, sensitive and simple in vivo biosensor to determine blood glucose levels has also gained considerable attention among researchers during the past few years. Recently, Lin and his colleagues revealed a glucose spectrophotometric colorimetric determination using V_2_O_5_ as GOD mimics, which monitored the changes in glucose levels in the brain of living rats in real time [45]. Using the measured absorbance, the change in the glucose concentration in the living and cooked brains could be observed in real time.

### 4.2. DNA Detection

DNA is the critical hereditary substance in almost all organisms and most diseases are highly related to genomic DNA damage and double-stranded DNA fragments [66]. Therefore, there has been ever-growing interest to develop sensitive assays for DNA detection. To date, the polymerase chain reaction (PCR)-based technique has been one of the most commonly employed methods for DNA sequence amplification and determination. However, it encounters complicated procedures and can be easily contaminated. With the fast development of nanotechnology and nanozyme-based detecting techniques, several GOD-mimicking nanomaterials have been tentatively explored for the analysis of low-concentration target DNA and DNA hybridization.

Upon interacting with biomolecules (e.g., protein DNA, RNA), the stability and GOD-like activity of nanomaterials will be tuned, which can be developed for the detection of various biomolecules. Interestingly, single-strand DNA (ssDNA) exhibited a much stronger affinity to the Au NPs than double-strand DNA (dsDNA). Therefore, when ssDNA interacts with Au NPs, the strong noncovalent binds greatly suppress the GOD-like activity of the Au NPs, whereas the weak interaction between the Au NPs and dsDNA only slightly perturbs the glucose oxidation process catalyzed by the Au NPs.

**Figure 7:**
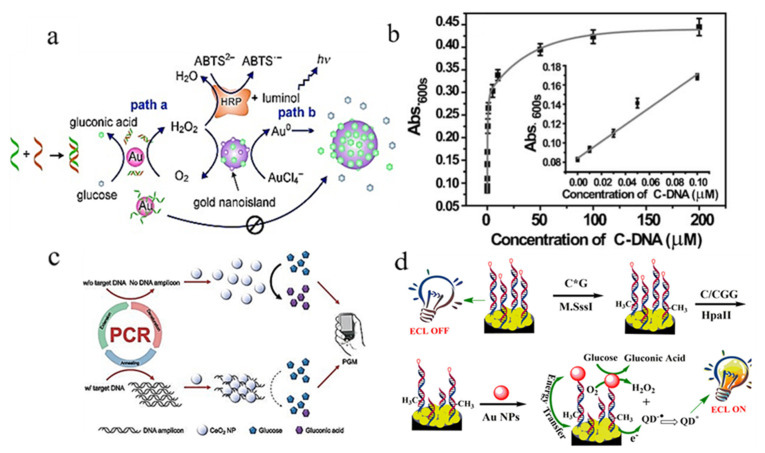
GOD-mimicking nanomaterials for the detection of DNA and DNA-related enzyme activity. (**a**) GOD-mimicking Au NPs for nanoplasmonic determination of DNA hybridization by coupling with an HRP-based colorimetric or chemiluminescent assay. Reprinted with permission from Ref. [67]. Copyright 2011, John Wiley & Sons. (**b**) Nanocomposites containing POD-mimicking V_2_O_5_ nanowires and GOD-mimicking Au NPs for target complementary DNA detection. Reprinted with permission from Ref. [68]. Copyright 2014, John Wiley & Sons. (**c**) GOD-mimicking nanoceria for personal glucose meter-based DNA detection. Reprinted with permission from Ref. [38]. (**d**) GOD-mimicking Au NPs for the sensitive detection of the methyltransferase activity. Reprinted with permission from Ref. [71]. Copyright 2016, American Chemical Society.

By taking advantage of this unique feature, Fan and his colleagues provided a quantitative determination of the target DNA or microRNAs (Figure 7a) [67]. Upon coupling with the HRP-based colorimetric or chemiluminescent assay, the limit of detection (LOD) of the target DNA was approx. 0.75 nM. Notably, natural HRP or POD-like nanomaterials can be combined into nanocomposites for a cascade reaction in DNA detection. For instance, nanocomposites containing POD-mimicking V_2_O_5_ nanowires and GOD-mimicking Au NPs can be used to detect the target complementary DNA and distinguish the disease-associated single-nucleotide polymorphism of DNA (Figure 7b) [68]. In addition, owing to the previously mentioned self-catalyzed activity of GOD-mimicking Au NPs, the enlarged size of Au NPs imparts the nanoplatform with localized nanoplasmonic properties, which can be exploited to visually identify DNA targets or DNA hybridization at the single nanoparticle level using dark-field illumination [20]. Subsequently, the GOD-mimicking Au NPs were assembled into the insulative gaps as an electrical biosensor for DNA hybridization detection [69]. With the addition of probe ssDNA, the GOD-like activity of Au NPs was completely blocked, and a negligible conductance response was observed in the microelectrode assays. In contrast, upon adding the target DNA, the obtained dsDNA were not adsorbed onto the surface of the Au NPs and the unmodified Au NPs maintained the remarkable GOD-like activity. As a result, the Au NPs were enlarged and connected the gap between the microelectrode assays, dramatically increasing the conductance. This label-free electrical assay can be used for clinical genetic analyses.

Recently, other nanomaterials instead of Au NPs were also developed for label-free DNA detection. For instance, the GOD-like activity of nanoceria can be inhibited by amplified DNA, and the amount of amplified DNA can be measured by the reduction in the glucose level (Figure 7c). Therefore, Park et al. designed a novel nanoceria-based personal glucose meter for the on-site read-out of the target DNA amplification in less than 5 min [38]. As the proof of concept, the developed personal glucose meter could be used for the detection of *E. coli* genomic DNA in a real serum sample down to 10 copies.

With the development of GOD-mimicking nanozymes for DNA detection, the simple and label-free detection strategy can be expanded to the measurement of some DNA-related enzyme activities [70,71]. For instance, GOD-mimicking Au nanomaterials were designed for the detection of the methyltransferase activity (Figure 7d) [71]. Briefly, dsDNA, including specific CpG dinucleotides, could be recognized and cut off by restricting endonuclease HpaII. The resulting dsDNA section with SH groups was combined with the GOD-mimicking Au NPs to enhance the electrochemiluminescence of the CdS quantum dots. However, the addition of CpG methyltransferase catalyzed the methylation of CpG dinucleotides and blocked the breakage of dsDNA. Therefore, based on the linear correlation between the electrochemiluminescence intensity and the CpG methyltransferase activity, a promising biocompatible platform for the methyltransferase activity was obtained with a LOD of 0.05 U mL^−1^.

### 4.3. Tumor Treatment

Anaerobic glycolysis, known as the Warburg effect, is one of the inherent hallmarks of cancer metabolism, which is characterized by high levels of glucose uptake and an increased conversion of glucose to lactose via the glycolytic pathway [72]. As a result, tumor tissues exhibit a mildly acid microenvironment and are highly sensitive to the fluctuation of the glucose content. Inspired by this feature, GOD have been used to cut off the nutrition source of cancer cells in tumor starvation therapy. Compared to natural GOD, GOD-mimicking nanomaterials offer a higher catalytic stability, easier modification and a lower manufacturing cost for tumor treatment.

Currently, GOD-mimicking nanomaterials applied in tumor therapy mainly include Au NPs and MnO_2_ NPs. On the one hand, they accelerate the consumption of tumorous glucose and reduce the energy supply to tumor cells, which selectively starves the tumors. On the other hand, H_2_O_2_ is produced at the same time when glucose is oxidized, which can be used as a precursor of molecule oxygen or highly toxic free radicals to participate in cascade therapy. In combination with the designability and multifunctionality of nanostructures, GOD-mimicking nanomaterials could be assembled with other components in the nanoplatforms, such as MOF, COF, mesoporous silica or hollow black TiO_2_ nanosphere, to achieve synergistic cancer therapy using photothermal therapy (PTT), photodynamic therapy (PDT) and sonodynamic therapy (SDT) [30,73,74].

**Figure 8:**
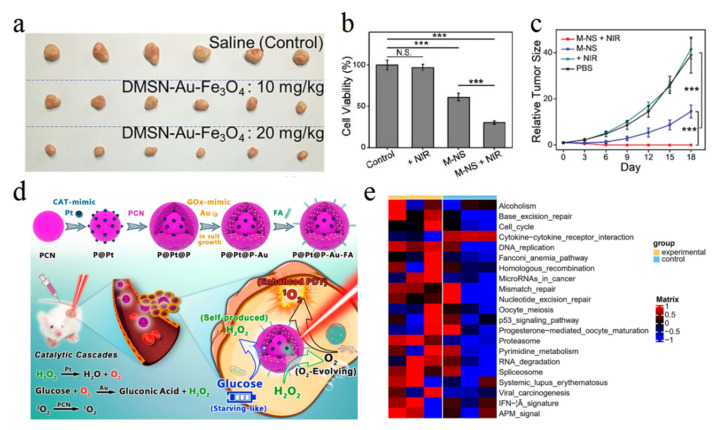
GOD-mimicking nanomaterials for tumor therapy. (**a**) Concentration-dependent tumor inhibition using a DMSN–Au–Fe_3_O_4_ nanoplatform. Reprinted with permission from Ref. [35]. Cell killing (**b**) and tumor growth inhibition (**c**) mediated by 2D MnO_2_ nanosheets-induced starvation therapy for synergistically enhanced photothermal therapy. Reprinted with permission from Ref. [12]. Copyright 2010, John Wiley & Sons. (**d**) Porphyrin MOF for catalytic cascades-enhanced cancer therapy. Reprinted with permission from Ref. [29]. Copyright 2019, American Chemical Society. (**e**) Nanozyme-engineered HABT-C with amplified sonodynamic therapeutic effects. Reprinted with permission from Ref. [28]. Copyright 2022, American Chemical Society. *** *p* < 0.001. N.S.—No significance.

In 2019, Shi, Chen and their colleagues first reported Au-based GOD-mimicking nanomaterials for nanocatalytic tumor therapy [35]. By immobilizing the GOD-mimicking Au NPs and POD-mimicking Fe_3_O_4_ NPs into the pore channels of the dendritic mesoporous silica NPs, the generated DMSN–Au–Fe_3_O_4_ nanoplatform triggered the tumor microenvironment (TME)-responsive cascade catalytic reaction for efficient tumor therapy (Figure 8a). The in situ grown Au NPs specifically catalyze the oxidation of glucose and produce H_2_O_2_ in the tumor site, which is further converted into highly active hydroxyl radicals under the catalysis of the magnetic Fe_3_O_4_ NPs and suppress the tumor growth. In the same year as an alternative paradigm of GOD-mimicking nanomaterials, 2D MnO_2_ nanosheets designed by Chen et al. were developed for effective cancer starvation therapy [12]. Both the cancer cells viability and tumor growth could be inhibited by the nanozyme-catalyzed depletion of intratumor glucose (Figure 8b,c). Specially, 2D MnO_2_ nanosheets have a high absorption in the NIR range, presenting a synergistic starvation-enhanced photothermal therapy both in vitro and in vivo.

In addition to photothermal therapy, GOD-mimicking nanomaterials could be combined with PDT and SDT. For instance, Wu et al. proposed an in situ catalytic cascade nanoreactor that consisted of a dual nanozyme-engineered porphyrin MOF, in which the GOD-mimicking Au NPs accelerated the depletion of intratumoral glucose for starvation therapy and the CAT-mimicking Pt NPs catalyzed the intratumoral/generated H_2_O_2_ into O_2_ for the alleviation of tumor hypoxia, consequently enhancing the O_2_-dependent PDT therapy of the nanoreactor (Figure 8d) [29]. Recently, a multifunctional platform based on Au NPs modified with a hollow black TiO_2_ nanosphere (HABT-C) with intrinsic multi-enzyme (GOD, CAT and POD) mimicking activities was developed for SDT to realize the reversion of tumor immunosuppression (Figure 8e) [28]. Under ultrasound irradiation, the enzyme-mimicking activity of HABT-C was improved by facilitating the electron–hole separation and the absorption of H_2_O and O_2_. Specifically, HABT-C exhibits a favorable inhibition of the immunosuppressive mediator expression, consequently amplifying the SDT efficiency. In a word, GOD-mimicking nanomaterials not only can be used for tumor starvation therapy but can combine other therapy modalities to realize synergistic tumor therapy via the regulation of the tumor microenvironment.

## 5. Summary and Perspectives

Glucose, a widely existing substance in nature, is the most important energy source for all living organisms. The research on the glucose conversion has great practical and biological significance. As one of the most important glucose-converting enzymes, glucose oxidase (GOD) provides a steady stream of energy for life by catalyzing the aerobic glucose oxidation. In addition, GOD-containing systems have been widely applied in industrial and biomedical applications. However, the intrinsic drawbacks of natural GOD, especially its poor stability under harsh and sophisticated physiological environments, restrict its performance and further applications. Fortunately, plenty of artificial nanomaterials have been recently discovered with a GOD-like activity and their catalytic efficiency, as well as catalytic selectivity, have been continuously optimized for diverse applications, varying from biosensing to disease treatment. Inspired by the catalytic generation of gluconic acid and H_2_O_2_, GOD-mimicking nanomaterials can be further combined with other natural enzymes or nanozymes for effective biocatalytic cascades, which will greatly enrich the potential biomedical applications of GOD. Despite the notable progress that has been made, the biomedical translations of GOD-mimicking nanomaterials still have many opportunities and challenges that need to be addressed.

### 5.1. Discovery of Novel GOD-mimicking Nanomaterials

As mentioned above, several nanomaterials, such as the Au NPs, nanoceria, MnO_2_, C_3_N_4_ NPs, have been discovered that possess a GOD-like activity. However, in comparation to the other nanozymes, the discovery of GOD-mimicking nanomaterials is still in its infancy and further exploration should be carried out to design or fabricate novel nanostructures to catalyze the oxidation of glucose.

### 5.2. Improvement of the GOD-like Activity

Although several nanomaterials have been discovered with a high GOD-like activity, the catalytic efficiency is still insufficient when compared to natural GOD, which greatly limits their biological applications. Therefore, in order to optimize the catalytic efficiency toward glucose oxidation, researchers have proposed a variety of strategies, including but not limited to increasing the reaction sites or the specific surface area, optimizing the structure and composition of the nanomaterials, regulating the optimal surrounding temperature and pH, and so on. However, the GOD-like activity of the reported nanostructures is still unsatisfactory in comparison to natural GOD. To maximize the catalytic activity toward glucose oxidation, bioinspired designs, such as state-of-the-art single atom technology, might be a promising approach for promoting the utilization efficiency of the catalytic sites.

### 5.3. Regulation of the Catalytic Selectivity

The selectivity of natural GOD to glucose is mainly derived from its unique spatial configuration. However, most of the reported GOD-mimicking nanozymes lacked spatial organization, which restricted the substrate glucose specificity. For example, glucose and fructose share similar chemical structures but have distinct biological functions. To impart the GOD-mimicking nanozymes with substrate selectivity, researchers designed a variety of nanostructures with reaction sites specific to glucose. In this regard, the surface modification of the specific binding pockets (e.g., molecularly imprinted polymers, well-arranged amino acid residues) or the chiral design of GOD-mimicking nanomaterials offers an alternative strategy.

### 5.4. Expansion of Practical Applications

GOD-based biosensors have been widely used in health, food and environmental areas. Compared to natural GOD, GOD-mimicking nanozymes with a considerable catalytic performance and a high durability have expansive application prospects for the detection of various analytes. Therefore, the product engineering should be conducted to meet the requirements of biological samples testing. In cancer treatment, the systematic biosafety evaluation of the designed GOD-mimicking nanozymes should be confirmed to provide a toxicologic basis for further practical biological applications. In addition, the potential applications of GOD-mimicking nanozymes have only recently been realized, and they need to be greatly expanded.

## Figures and Tables

**Table 1:** Representative nanocarriers to support GOD-mimicking Au NPs and their applications.

Nanocarrier	Size of Au NPs	Application	Ref
Mesoporous silica	2.1 nm	Cascade catalysis	[23]
Activated carbon	7–8 nm	Production of gluconate	[24]
Fe_3_O_4_	4.2 nm	Cascade catalysis	[25]
Al_2_O_3_	1.9–16.6 nm	Production of gluconate	[26]
Graphene	\	Cascade catalysis	[27]
TiO_2_	\	Tumor therapy	[28]
MOF	2 nm	Cascade catalysis	[29]
COF	3 nm	Cascade catalysis	[30]

## Data Availability

Not applicable.
